# Intraindividual changes of dipeptidyl peptidase-IV in peripheral blood of patients with rheumatoid arthritis are associated with the disease activity

**DOI:** 10.1186/s12891-015-0707-y

**Published:** 2015-09-09

**Authors:** Lucie Sromova, Petr Busek, Liliana Sedova, Aleksi Sedo

**Affiliations:** Laboratory of Cancer Cell Biology, Institute of Biochemistry and Experimental Oncology of the First Faculty of Medicine, Charles University in Prague, U Nemocnice 5, 128 53 Prague 2, Czech Republic; Institute of Rheumatology, Na Slupi 4, 128 50 Prague, Czech Republic

## Abstract

**Background:**

Dipeptidyl peptidase-IV (DPP-IV) is suggested to contribute to the pathogenesis of several autoimmune diseases. The aim of this study was to evaluate the association of DPP-IV presence in blood plasma and mononuclear cells with the disease activity in rheumatoid arthritis (RA).

**Methods:**

Patients with active RA (*n* = 27) were examined at the study enrolment and a follow-up examination was performed after the regression of the joint effusions and at least 6 months after the first investigation. The control group comprised patients with a noninflammatory joint disease, i.e. osteoarthritis (OA; *n* = 15). The DPP-IV-like enzymatic activity was measured by a kinetic fluorimetric method, the concentration of DPP-IV in the blood plasma was determined using ELISA and the expression of DPP-IV in leukocytes was assayed by flow cytometry.

**Results:**

Blood plasma DPP-IV-like enzymatic activity (median ± SD 220.15 ± 83.6 pkat/mL in RA vs. 376.9 ± 144.9 pkat/mL in OA, *p* < 0.001) and concentrations (median ± SD 465.1 ± 215.6 ng/mL in RA vs. 953.3 ± 368.4 ng/mL in OA, *p* < 0.001) were lower in patients with active RA compared to OA. In RA patients, the blood plasma DPP-IV-like enzymatic activity negatively correlated with the CRP concentration (*r* = −0.39, *p* = 0.044). No significant differences were observed in the DPP-IV-like enzymatic activity and DPP-IV expression in blood mononuclear cells between the RA and OA groups. At follow-up, 18 RA patients had a less active disease as demonstrated by an improved DAS28 score. In this group, comparison of the entry and the follow-up values in individual patients revealed an increase of the blood plasma DPP-IV-like enzymatic activity (median ± SD 141 ± 46 % of the patient’s entry values, *p* = 0.011) and DPP-IV concentration (median ± SD 168 ± 25 %, of the patient’s entry values, *p* = 0.033). In contrast to the blood plasma, the DPP-IV expression in blood mononuclear cells was reduced in these patients as evidenced by a decrease in the cell surface DPP-IV-like enzymatic activity as well as the median fluorescence intensity of DPP-IV staining in lymphocytes (median ± SD 66 ± 56 %, *p* = 0.018 and 63 ± 31 % of the patient’s entry values, *p* = 0.005, respectively).

**Conclusions:**

The association between RA activity and the changes in blood plasma and blood mononuclear cell DPP-IV in individual patients supports the possible relationship of DPP-IV to RA pathophysiology.

## Background

Dipeptidyl peptidase-IV (DPP-IV, CD26, EC 3.4.14.5) is a plasma membrane peptidase that selectively cleaves an N-terminal dipeptide from peptides with a proline or alanine residue in the penultimate position. DPP-IV is widely expressed on epithelial and endothelial cells and a soluble form can be found in body fluids including blood plasma, where it represents at least 95 % of the DPP-IV-like enzymatic activity [1]. In the immune system, DPP-IV is expressed preferentially by the CD4+CD45RO+ memory T cells, is associated with the Th1 response and its expression is upregulated following T cell activation [2, 3]. In contrast, DPP-IV is low or undetectable on B-cells, NK cells and monocytes in healthy adults [4]. DPP-IV is well known as a T cell co-stimulatory molecule involved in the T cell activation and proliferation [5]. Some of the DPP-IV effects are executed by its hydrolytic activity (processing of cytokines, chemokines and neuropeptides), while some others are mediated by non-hydrolytic interactions (adenosine deaminase binding, interaction with the tyrosine-phosphatase CD45) [5–7]. In *in vitro* studies, some of the DPP-IV inhibitors were demonstrated to suppress the production of various cytokines and enhance the production of the suppressive cytokine transforming growth factor beta [8].

Changes in the expression and/or the blood plasma concentration of DPP-IV are associated with several diseases including rheumatoid arthritis (RA) [5], an autoimmune inflammatory disorder characterized by synovial inflammation leading to cartilage destruction as well as systemic manifestations. The pathogenesis of RA has not been clearly elucidated so far, but involves an interplay of predisposing genetic factors, sex hormones and possibly an infectious or another immunity triggering agent that ultimately lead to an inappropriate activation of the immune system and perpetuating inflammation [9]. Given the role of DPP-IV in T cell activation [10] and its ability to cleave numerous proinflammatory peptides involved in the pathogenesis of RA [5], several studies examined its possible significance in the disease progression. The results of these studies, including ours, have so far revealed (although variably) a decreased DPP-IV-like enzymatic activity in the mononuclear cells isolated from the synovial fluid of RA patients [11, 12] as well as decreased blood plasma DPP-IV enzymatic activity/concentration [12–14]. Higher DPP-IV expression was reported in blood mononuclear cells and especially CD4+ T lymphocytes in RA patients with high disease activity compared to healthy controls [15–18]. The literature data on the association of these changes with the disease activity are inconsistent [12, 14, 18, 19]. Furthermore, it is currently unclear whether the changes of DPP-IV in blood plasma and peripheral mononuclear cells relate to the disease activity on the intraindividual basis.

## Methods

27 patients with RA diagnosed according to the standard criteria of the American College of Rheumatology were examined during the active phase of their disease with joint effusion (active RA), 15 patients with osteoarthritis served as controls (Table 1). RA disease activity was evaluated using the DAS28 score based on the blood plasma concentration of the C-reactive protein (CRP), patient-assessed visual analogue scale (VAS) and swollen and tender joint counts (http://www.das-score.nl/). The follow-up examination in RA patients was performed after joint effusion regression and at least 6 months after the first investigation. Intraindividual changes of the studied parameters were evaluated in patients with at least a moderate improvement of the disease activity as defined by the change of the DAS28 score (a decrease in DAS28 > 0.6 if the current value was <5.1 or a decrease in DAS28 > 1.2 if the current value was >5.1). The study was approved by the Institutional ethics committee of the Institute of Rheumatology in Prague under the code EK-C327LS/06 and was conducted in accordance with the Declaration of Helsinki. All patients signed informed consent.

**Table 1:** Clinical characteristics of the patients included in the study

	Rheumatoid arthritis (*n* = 27)	Ostheoarthritis (*n* = 15)
Age	59 ± 13	62 ± 11
Male/Female	7/20	6/9
Disease duration (years)	12.5 ± 12.4	–
Swollen joint counts (0–28)	9 ± 6.3	–
Tender joint counts (0–28)	11 ± 6.7	–
DAS 28	5.8 ± 1.1	–
ESR (mm/h)	56 ± 28.2	10 ± 6.4
CRP (mg/L)	44 ± 26	5 ± 4

*DAS 28* Disease activity score, *CRP* C-reactive protein, *ESR* Erythrocyte sedimentation rate. The values are medians ± SD

Peripheral blood was collected under sterile conditions into BD Vacutainer (BD Biosciences, USA) with sodium heparin as an anticoagulant. Blood mononuclear cells (BMNC) were isolated by discontinuous Ficoll-Paque density centrifugation in the “cell preparation tube” with sodium heparin (CPT, BD Biosciences, USA). Isolated BMNC were counted on a cell counter Z2 (Beckman Coulter, USA); there were more than 95 % of viable cells as determined by trypan blue exclusion.

The DPP-IV-like enzymatic activity in the heparinized blood plasma and in BMNC was measured by a continuous rate fluorimetric assay with 7-(glycyl-prolylamido)-4-methylcoumarin (Bachem, Switzerland, final concentration 50 μmol/L), as a substrate at pH 7.5 and 37 °C. The release of 7-amino-4-methylcoumarin was monitored at excitation and emission wavelengths of 380 and 460 nm, respectively (Spectrofluorimeter Perkin Elmer LS50B). The cell surface DPP-IV-like enzymatic activity in BMNC was determined as the activity of viable cells, the total DPP-IV-like enzymatic activity was measured under the same conditions after permeabilization of the cells with 0.1 % Triton X-100 [12].

Immunophenotypization of the BMNC was performed by a flow cytometer FACS Canto (BD Biosciences, USA) with the Diva software for acquisition and FlowJo (TreeStar Inc.) for data evaluation. 50 μL of the peripheral blood was incubated for 30 min at room temperature with mouse anti-CD3-PerCP, anti-CD4-APC, anti-CD8-APC-Cy7 and anti-CD14-PE-Cy7 (all from BD Biosciences), rat anti-DPP-IV/CD26-FITC (RD systems). All antibodies were used in the titre of 1:20. Lymphocyte subsets were identified by gating analysis and fluorescence profiles were obtained for 10 000 cells in each sample. Median fluorescence intensity (MFI) of DPP-IV/CD26 expression in lymphocytes was calculated as a ratio of the median fluorescence intensity of the DPP-IV/CD26 positive and negative lymphocyte populations.

Blood plasma DPP-IV concentration was determined by the sandwich enzyme-linked immunosorbent assay (“Duo set” ELISA kit – DY1180, RD Systems, UK), according to the manufacturer’s instructions. A microplate reader Sunrise (Tecan, Switzerland) was used to measure the absorbance at 450 nm, a wavelength correction was performed at 570 nm.

The Statistica 12 software (StatSoft, Inc., USA) was used for statistical analyses. The Wilcoxon pair test and the Mann–Whitney *U* test were used as appropriate. Correlations were analyzed by the Spearman’s correlation coefficient.

## Results

### DPP-IV in patients with active rheumatoid arthritis

**Fig. 1:**
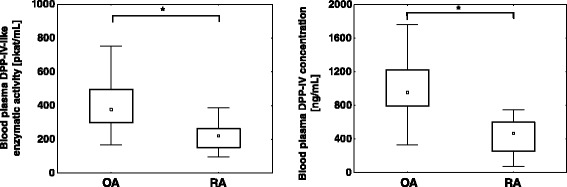
Blood plasma DPP-IV-like enzymatic activity and concentration in RA and OA patients. In the box plots, the medians are depicted as small squares, interquartile range (25–75th centile) as boxes and bars extend from the minimum to maximum values. *p < 0.001, Mann–Whitney *U* test

**Fig. 2:**
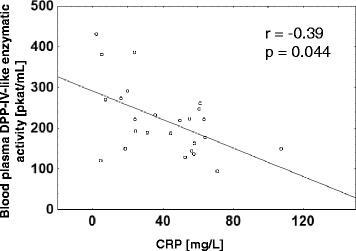
Negative correlation between the blood plasma DPP-IV-like enzymatic activity and CRP concentration in RA patients. Spearman’s correlation coefficient is shown

Compared to patients with osteoarthritis (OA), rheumatoid arthritis (RA) patients recruited in the active phase of their disease exhibited significantly lower blood plasma DPP-IV-like enzymatic activity (median ± SD 220.15 ± 83.6 pkat/mL in RA vs. 376.9 ± 144.9 pkat/mL in OA, *p* < 0.001) and blood plasma DPP-IV concentration (median ± SD 465.1 ± 215.6 ng/mL in RA vs. 953.3 ± 368.4 ng/mL in OA, *p* < 0.001) (Fig. 1). The blood plasma DPP-IV-like enzymatic activity correlated with DPP-IV concentration determined by ELISA (*r* = 0.83, *p* < 0.001). In RA patients, there was a negative correlation between the DPP-IV-like enzymatic activity and the CRP concentration (Fig. 2). These results were consistent with the previously published data, including ours [5, 12, 20].

Neither the DPP-IV-like enzymatic activity in blood mononuclear cells nor the DPP-IV expression in lymphocytes determined by flow cytometry were significantly different in patients with active RA compared to OA (data not shown).

### Intraindividual changes of DPP-IV in blood plasma and in blood mononuclear cells parallel disease activity in RA

**Fig. 3:**
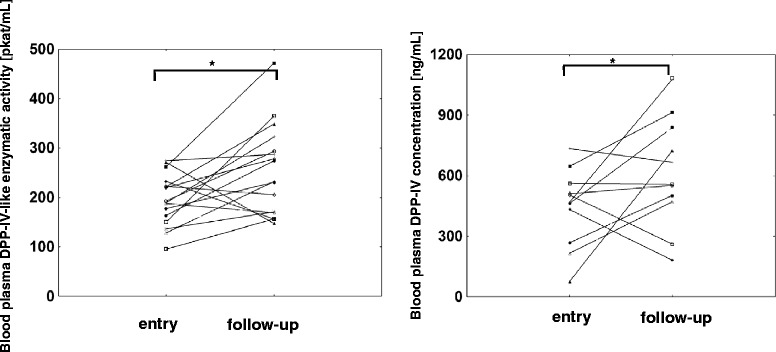
Blood plasma DPP-IV in RA patients with clinical improvement of the disease. Blood plasma DPP-IV-like enzymatic activity (*n* = 18) and DPP-IV concentration (*n* = 13) were determined in patients exhibiting at least a moderate improvement of the disease, individual entry and follow-up values are depicted. *p < 0.05, Wilcoxon pair test

To evaluate whether the changes in the blood plasma DPP-IV reflect disease activity in individual patients, a follow-up evaluation was performed in 18 patients with RA during a less active phase of their disease as defined by the regression of joint effusion and improved DAS28 score compared to the entry values. The disease activity decreased (Table 2) as evidenced by the reduction of the markers of inflammation CRP and ESR. In 13 patients (72 %), this improvement was associated with a rise of DPP-IV-like enzymatic activity in the blood plasma of at least 20 % compared to the individual patient’s entry values. The enzymatic activity remained unchanged in 3 (17 %) and decreased in 2 (11 %) patients. Overall, the blood plasma DPP-IV-like enzymatic activity rose to 141 ± 46 % (median ± SD, *p* = 0.011) compared to the entry values of individual patients. A similar increase to 168 ± 25 % (median ± SD, *p* = 0.033) of the patient’s entry values was observed for the blood plasma DPP-IV concentration as determined by ELISA. The changes of these parameters in individual patients are shown in Fig. 3. Despite this observed increase of the blood plasma DPP-IV, the median of the values in the RA patients in the less active state of the disease still remained approximately 30 % below the levels in the OA patients (data not shown), most likely due to the background of the RA disease activity.

**Table 2:** Clinical characteristics of the RA patients exhibiting at least moderate improvement at the follow-up examination

	Entry values	Follow-up
Age	60 ± 16	61 ± 16
Male/Female	5/13	5/13
Disease duration (year)	4.5 ± 13.3	5.5 ± 13.5
Swollen joint count (0–28)	9 ± 6	2 ± 3
Tender joint count (0–28)	11.5 ± 6.5	2.5 ± 6.6
DAS 28	5.66 ± 0.97	3.49 ± 1.22
ESR (mm/h)	56 ± 27.7	30 ± 24.1
CRP (mg/L)	51.08 ± 24.49	7.31 ± 19.13

*DAS 28* Disease activity score, *CRP* C-reactive protein, *ESR* Erythrocyte sedimentation rate. The values are medians ± SD

**Fig. 4:**
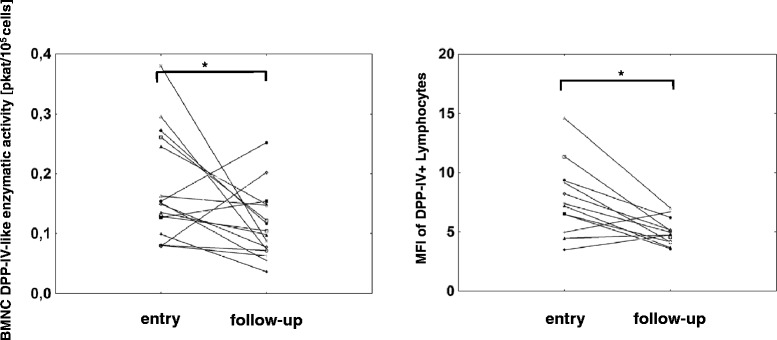
DPP-IV in blood mononuclear cell (BMNC) in RA patients with clinical improvement of the disease. Cell surface DPP-IV-like enzymatic activity (*n* = 18) and the median fluorescence intensity (MFI) of DPP-IV in lymphocytes (*n* = 14) were determined in patients exhibiting at least a moderate improvement of the disease, individual entry and follow-up values are depicted. *p < 0.05, Wilcoxon pair test

In contrast to the dynamics of the blood plasma DPP-IV, an inverse trend was seen in the BMNC (Fig. 4). The plasma membrane as well as the total DPP-IV-like enzymatic activity in BMNC decreased to 66 ± 56 % (median ± SD, *p* = 0.018) and 52 ± 64 % (*p* = 0.005), respectively, of the entry values of individual patients. Using flow cytometry, DPP-IV/CD26 was detected in lymphocytes and only exceptionally a very low positivity was observed in monocytes (data not shown). The percentage of CD26 positive lymphocytes was statistically significantly decreased compared to the individual patient entry values (*p* = 0.029, data not shown). Larger changes were observed in the quantity of DPP-IV/CD26 expression as determined by median fluorescence intensity, which was decreased to 63 ± 31 % of the patient’s entry values (median ± SD, *p* = 0.005).

Taken together, the attenuation of RA disease activity was accompanied by a rise of the blood plasma DPP-IV and a reduction of DPP-IV expression in BMNC in the majority of patients. At a cut-off level of a 20 % change in comparison to the entry values for DPP-IV enzymatic activity, 45 % of the patients exhibited both of these changes concurrently.

## Discussion

Dipeptidyl peptidase-IV (CD26) is a T lymphocyte costimulatory molecule and is also involved in the proteolytic regulation of several proinflammatory mediators [5]. Numerous studies have therefore explored the role of DPP-IV in autoimmune and inflammatory diseases, including systemic lupus erythematosus, multiple sclerosis, inflammatory bowel disease and RA [14, 21–25]. Decreased DPP-IV in blood plasma in comparison with healthy controls was described in patients with systemic lupus erythematosus, inflammatory bowel disease and multiple sclerosis [5, 23, 24, 26]. In contrast with blood plasma, several authors found higher frequency of DPP-IV positive blood cells compared to healthy controls in inflammatory bowel disease and multiple sclerosis patients [23, 25]. In addition, there was a correlation of the frequency of CD4+DPP-IV high+ cells with the clinical severity in patients with multiple sclerosis [3, 26], while reduced levels of the DPP-IV expression in BMNC were observed in highly active systemic lupus erythematosus [24].

RA patients exhibit lower blood plasma DPP-IV-like enzymatic activity compared to the controls [14, 20, 27, 28]. Patients with a noninflammatory joint disease (osteoarthritis) were used as a control group in our current study. We demonstrate that both the DPP-IV-like enzymatic activity and DPP-IV concentration were almost 50 % lower in RA compared to patients with osteoarthritis. Cuchacovich et al. suggested that the decrease of DPP-IV enzymatic activity in blood plasma may be a consequence of the lower specific enzymatic activity of DPP-IV in RA patients compared to the healthy subjects caused by its hypersialylation [29]. Our data in agreement with other reports [13, 19, 20] show that the lower enzymatic activity observed in RA patients is in large part caused by the decreased blood plasma concentration of the DPP-IV protein.

In our study, we observed neither a significant difference in the proportion of DPP-IV positive lymphocytes nor in the intensity of DPP-IV/CD26 expression in lymphocytes between RA and osteoarthritis patients. Moreover, the DPP-IV-like enzymatic activity in blood mononuclear cells was similar in RA and OA patients (data not shown), which was in line with our previously published data on an independent patient cohort [12]. Increased DPP-IV/CD26 expression on blood mononuclear cells of RA patients in comparison to healthy individuals was proposed by some authors. Muscat et al. [15] suggested that higher RA disease activity was associated with increased DPP-IV expression in peripheral blood T cells, while patients with less active disease had DPP-IV expression comparable to healthy subjects. In addition, Ellingsen et al. observed a significant increase of the DPP-IV/CD26 antigen expression in CD4+ T cells only in patients with chronic (median disease duration 11.5 years) rheumatoid arthritis [16], while in RA patients early after diagnosis (<6 months), there were no differences in comparison to healthy controls [30]. The direct comparison of the so far published studies is problematic due to the variance of the disease activity typically occurring within the experimental groups as well as different methodologies used for the disease activity assessment, different control groups (osteoarthritis patients vs. healthy individuals) and variable duration of the illness.

Our previously published data showed a negative correlation between the DPP-IV-like blood plasma enzymatic activity and CRP [12]. Cordero et al. [19] described the inverse correlation between the blood serum DPP-IV concentration and the number of swollen joints, but did not observe differences in the blood serum DPP-IV concentration in the groups of patients with active as compared to inactive RA. Similarly, Ulusoy et al. [13] did not find a significant association between DPP-IV blood serum concentration and RA disease activity. Higher expression of DPP-IV in T cells was detected in patients with active RA as compared to the less active RA [15, 17]. The main focus of our current study was to extend and strengthen the so far available evidence of the association of DPP-IV with the RA disease activity by analyzing the intraindividual dynamics of DPP-IV associated with the disease improvement. During our study, the patients received various types of therapy based on the clinical judgment of the attending physician (glucocorticoids, methotrexate, anti-TNF alpha and anti-CD20 antibodies, leflunomide, sulfasalazine and their combinations). Despite this heterogeneity of the administered treatment, we observed consistent and significant intraindividual increase in the blood plasma DPP-IV enzymatic activity and concentration in follow-up examinations in patients with decreased RA activity. An increase of the DPP-IV enzymatic activity accompanied by a shift from acidic to more neutral glycoforms of the circulating DPP-IV was previously observed in RA patients with clinical improvement after anti TNF alpha therapy [31].

In addition to the changes in the levels of the circulating DPP-IV, our study suggests that both the DPP-IV-like enzymatic activity in BMNC and its expression in lymphocytes are decreased along with an improvement of the disease in individual patients. The intraindividual decrease of the DPP-IV/CD26 antigen expression was most notable in the CD4+ T cells (*p* = 0.055, data not shown) which is in agreement with the reported dominant presence of DPP-IV/CD26 in this lymphocyte subpopulation in RA patients [15].

The consistent increase of the plasma DPP-IV and the decrease of the DPP-IV expression on T cells observed in the majority of patients with the disease improvement in our study suggest the role of DPP-IV in RA pathophysiology. However, since we observed the simultaneous occurrence of both of these changes only in 45 % of the improved patients, it remains currently unclear, whether they are directly related to each other or whether they are part of different biological processes occurring during the disease improvement.

### Limitations of the study

Although the effects of the administered treatment and duration of the disease on the plasmatic and T cell DPP-IV are largely unknown, the heterogeneity of our RA patient cohort with regard to these variables may represent a possible limitation of our study.

## Conclusions

Blood plasma DPP-IV enzymatic activity and concentration are lower in RA patients during the active phase of the disease compared to the noninflammatory arthritis (osteoarthritis) patients. In addition, our intraindividual comparison demonstrates elevation of the blood plasma DPP-IV and a decrease of DPP-IV/CD26 in peripheral blood mononuclear cells associated with the clinical improvement in the majority of RA patients. These results further support the possible role of DPP-IV in the pathophysiology of RA.

## Abbreviations

BMNC: Peripheral blood mononuclear cells
CRP: C-reactive protein
DAS28: Disease activity score 28
DPP-IV: Dipeptidyl peptidase-IV
ELISA: Enzyme-linked immunosorbent assay
ESR: Erythrocyte sedimentation rate
MAb: Monoclonal antibody
MFI: Median fluorescence intensity
OA: Osteoarthritis
RA: Rheumatoid arthritis
VAS: Visual analogue scale
